# Network Pharmacology-Based Investigation and Experimental Exploration of the Antiapoptotic Mechanism of Colchicine on Myocardial Ischemia Reperfusion Injury

**DOI:** 10.3389/fphar.2021.804030

**Published:** 2021-12-16

**Authors:** Yuanjun Tang, Chenyang Shi, Yingyi Qin, Shuowen Wang, Hui Pan, Ming Chen, Xuemei Yu, Yuefen Lou, Guorong Fan

**Affiliations:** ^1^ Department of Clinical Pharmacy, Shanghai General Hospital, Shanghai Jiaotong University School of Medicine, Shanghai, China; ^2^ Department of Health Statistics, Naval Medical University, Shanghai, China; ^3^ Department of Pharmacy, Shanghai Fourth People’s Hospital Affiliated to Tongji University School of Medicine, Shanghai, China

**Keywords:** colchicine, myocardial ischemia reperfusion injury, network pharmacology, experimental verification, apoptosis

## Abstract

**Background:** The beneficial effects of colchicine on cardiovascular disease have been widely reported in recent studies. Previous research demonstrated that colchicine has a certain protective effect on ischemic myocardium and has the potential to treat myocardial ischemia reperfusion injury (MIRI). However, the potential targets and pharmacological mechanism of colchicine to treat MIRI has not been reported.

**Methods:** In this study, we used network pharmacology and experimental verification to investigate the pharmacological mechanisms of colchicine for the treatment of MIRI. Potential targets of colchicine and MIRI related genes were screened from public databases. The mechanism of colchicine in the treatment of MIRI was determined by protein-protein interaction (PPI), gene ontology (GO) and Kyoto Encyclopedia of Genes and Genomes (KEGG) pathway enrichment analysis. Additionally, we evaluated the effect of colchicine on H9C2 cell activity using CCK-8 assays, observed the effect of colchicine on H9C2 cell apoptosis via flow cytometry, and further verified the expression of key targets after colchicine treated by Western blot.

**Results:** A total of 626 target genes for colchicine and 1549 MIRI disease targets were obtained. 138 overlapping genes were determined as potential targets of colchicine in treating MIRI. the PPI network analysis demonstrated that the targets linked to MIRI were ALB, TNF, ACTB, AKT1, IL6, TP53, IL1B, CASP3 and these targets showed nice affinity with colchicine in molecular docking experiments. The results of GO analysis and KEGG pathway enrichment demonstrated that the anti-MIRI effect of colchicine involves in apoptotic signaling pathway. Further tests suggested that colchicine can protect H9C2 cell from Hypoxia/Reoxygenation (H/R) injury through anti-apoptotic effects. Western blot results demonstrated that colchicine can inhibited MIRI induced apoptosis of H9C2 cell by enhancing the decreased levels of Caspase-3 in myocardial injure model induced by H/R and activating the PI3K/AKT/eNOS pathway.

**Conclusions:** we performed network pharmacology and experimental evaluation to reveal the pharmacological mechanism of colchicine against MIRI. The results from this study could provide a theoretical basis for the development and clinical application of colchicine.

## Introduction

Acute Myocardial Infarction is the necrosis of myocardial tissue caused by acute or persistent ischemia and hypoxia of coronary arteries, which seriously threatens the lives of patients (Castro-Dominguez et al., 2018; Gulati et al., 2020). The most effective treatment for reducing the area of myocardial infarction is reperfusion timely (Bajaj et al., 2015; Toldo and Abbate, 2018). However, after restoring blood supply to the damaged myocardial tissue, many patients suffered more severe damage to the heart structure, more disordered energy metabolism of myocardial cells and even some irreversible damage such as re-enlargement of myocardial infarction area, life-threatening arrhythmia and so forth, therefore, we called it myocardial ischemia reperfusion injury (MIRI) (Buja, 2005; Marchant et al., 2012). Studies have revealed that the physiological mechanisms of MIRI are apoptosis, calcium overload, oxidative stress, autophagy, inflammation, endoplasmic reticulum stress, mitochondrial disorders, etc (Walters et al., 2012; Munzel et al., 2017). Previous research found that cardiomyocyte apoptosis is the core factor of MIRI, and about 50% of MIRI is related with cardiomyocyte apoptosis (Buja, 2005). Myocardial ischemia triggers the process of cardiomyocyte apoptosis, and reperfusion further aggravates the degree of this process, which would result in malignant arrhythmia, heart failure and even sudden cardiac death (Nagai et al., 2010; Fujiwara et al., 2019). Myocardial ischemia and hypoxia can induce the expression of a variety of apoptosis-related factors and inflammatory factors, which could promote abnormal apoptosis of myocardial cells (Yao et al., 2020). As an important factor affecting the progression of disease, the imbalance of myocardial cell apoptosis is also an important regulatory pathway for drugs to play a therapeutic role. Unfortunately, at present, there are few effective medicines for the treatment of MIRI in clinical practice.

Colchicine, which is a zhuophenone alkaloid extracted from the bulb of *Colchicum autumnale*, has extensive anti-inflammatory, immunosuppressive and strong anti-fibrosis effects, it is the first-line drug for the treatment of acute gout attack (Downing, 2000; Terkeltaub, 2009; Roubille et al., 2013). A retrospective study suggested that the use of colchicine in gout patients can reduce the incidence of acute myocardial infarction (Crittenden et al., 2012). Studies have reported that STEMI patients with chest pain less than 12 h were randomly treated with colchicine or placebo for 5 days after percutaneous coronary intervention (PCI), the CK-MB level in the colchicine group was declined and the area of infarction decreased significantly (Deftereos et al., 2015). The COLCOT study have demonstrated that colchicine can reduce the risk of recurring myocardial ischemic in patients after acute myocardial infarction and it has good safety in low dose (Tardif et al., 2019). What’s more, the earlier you start using colchicine after acute myocardial infarction, the greater the benefit (Bouabdallaoui et al., 2020). Above all, colchicine has a certain protective effect on ischemic myocardium and has the potential to treat MIRI, but its mechanism is still unclear.

**FIGURE 1:**
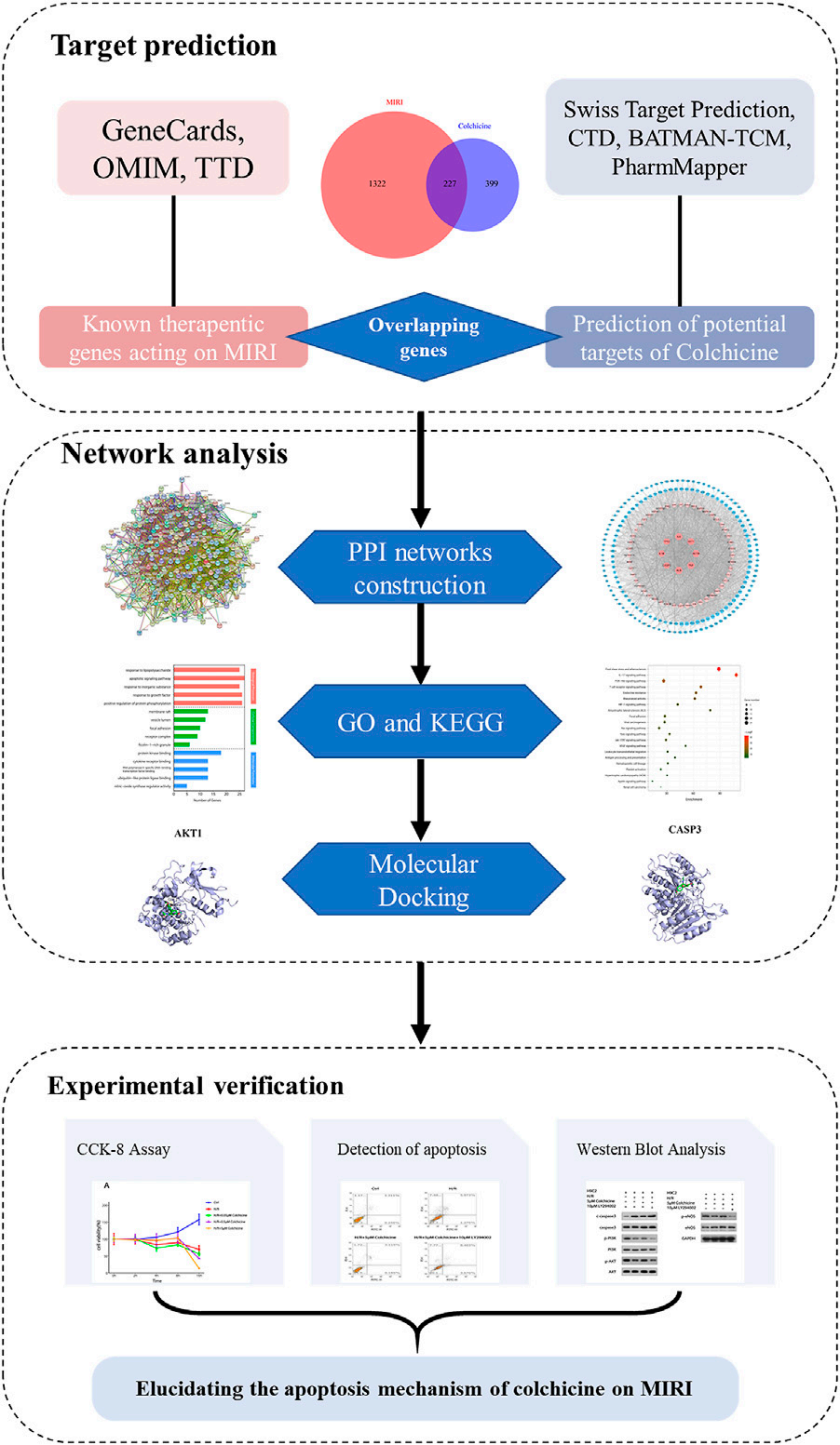
The workflow of action mechanism of Colchicine on treating MIRI in this study.

Network pharmacology is developed based on the theories of systems biology, network biology, multidirectional pharmacology and omics. It reveals the interaction mechanism between the body and disease from the aspects of protein, molecule and gene, and provides a basis for the development and mechanism research of multi-target drugs (Hopkins, 2007; Huang et al., 2014; Zhang et al., 2017). This study screened the potential targets and functional characteristics of colchicine in the treatment of MIRI through network pharmacology. The colchicine multi-target network was constructed, and the key targets were enriched. In addition, the key targets were identified by experimental verification to further confirm the effect of colchicine on related signal pathways. The process of network pharmacology analysis is shown in Figure 1. The main purpose of this study is to explore the therapeutic targets and key signal pathways of colchicine in treatment of MIRI, and provide potential strategies for disease treatment.

## Material and Methods

### Prediction of Potential Targets of Colchicine

The 2D molecular structure and PubChem CID of Colchicine were obtained from PubChem (https://pubchem.ncbi.nlm.nih.gov/, update time:2021-02-04), which is the world’s largest database of free access to chemical information. Swiss Target Prediction (http://www.swisstargetprediction.ch, update time:2019) (Daina et al., 2019), Comparative Toxicogenomics database (CTD, http://ctdbase.org/about/, update time: 2021-09-02) (Davis et al., 2019), BATMAN-TCM database (http://bionet.ncpsb.org.cn/batman-tcm/, update time: 2020-07-29) (Liu et al., 2016) and PharmMapper (http://lilab-ecust.cn/pharmmapper/, update time: 2017) (Wang et al., 2017) were used to obtain the targets name, gene name, UniProt ID and FIT score of colchicine. Then the names of target genes were converted into the official symbols using UniProt database (http://www.uniprot.org/, update time: 2021-04) (UniProt, 2019) and types was selected to “Homo sapiens”.

### Acquisition of MIRI Related Genes

The therapeutic targets for MIRI were collected from the Online Mendelian Inheritance in Man (OMIM, http://www.omim.org/, update time: 2021-09-02) (Amberger et al., 2015), Therapeutic Target database (TTD, https://db.idrblab. org/ttd/, update time: 2021-09-29) (Wang et al., 2020) and GeneCards (https://www.genecards.org/, update time: 2021-09-13) (Fishilevich et al., 2016). The MIRI was used as the searching keyword and the putative targets of colchicine and the known therapeutic targets on MIRI amalgamated.

### Protein-Protein Interaction (PPI) Networks Construction

The STRING database is a database of known and predicted protein-protein interaction. The potential targets of colchicine in the treatment of MIRI were put into STRING V11.5 (https://string-db.org/cgi/input.pl, update time: 2021) (Szklarczyk et al., 2017) to build the PPI network interaction. Cytoscape V3.8.2 (http://www.cytoscape.org/, update time: 2020-10-29) (Shannon et al., 2003) were used to construct and visualize the PPI network. The topological properties of PPI network were examined by 'Network analyzer' a cytoscape plugin app. Nodes in the network are connected by edges if there are regulatory relations. The parameters of “Degree”, “Average Shortest Path Length”, “Betweenness Centrality”, “Closeness Centrality”, “Clustering Coefficient” were estimated, and the value of “Degree” was used as a reference for the importance of the target.

### Gene Ontology (GO) and Kyoto Encyclopedia of Genes and Genomes (KEGG) Pathway Enrichment Analysis

Metascape (http://metascape.org/gp/index.html, update time: 2021-10-18) (Zhou et al., 2019) was used to conducted GO enrichment analysis and KEGG pathway enrichment analysis. The operation is simply to copy and paste the core genes in the Metascape’s gene list and then select the species of Homo sapiens. In the Enrichment section of the analysis page, KEGG Pathways, GO Molecular Functions, GO Biological Processes and GO Cellular Components were selected for enrichment analysis of KEGG Pathways, GO Molecular Functions, GO Biological Processes and GO Cellular Components respectively. The visualization of the important enrichment results of GO and KEGG pathways was implemented by “ggplot2” package of R version 4.0.2 software (update time: 2020-06-22).

### Molecular Docking

The 3D structure of the target protein was downloaded from the PDB database (https://www.rcsb.org/). The target protein is processed by removing ligand and water motifs and adding hydrogen using AutoDockTools-1.5.6 (update time: 2014-09-17) (Trott and Olson, 2010). The protein receptors and ligand were converted to PDBQT format. Finally, AutoDock vina software will be used for molecular docking, and the lowest free energy model is selected for visual analysis by using PyMOL. The docking score is carried out for each docking. The smaller the docking score, the better the docking result. Docking score < -6 indicates good binding affinity.

### Experimental Chemicals and Reagents

Fetal bovine serum (FBS), phosphate buffered saline (PBS), Krebs-Ringer Bicarbonate buffer, penicillin-streptomycin, trypsin-EDTA and dulbecco modified eagle medium (DMEM) were purchased from GIBCO (Grand Island, NY, United States). Protease inhibitor, RIPA Lysis buffer and BCA protein assay kit were obtained from Beyotime Biotechnology (Shanghai, China). Cell Counting Kit-8 (CCK8) detection kit was obtained from the BBI Life Sciences company (Shanghai, China). Annexin V-FITC/PI apoptosis kits were purchased from YEASEN biological technology company (Shanghai, China). Polyvinylidene fluoride (PVDF) membrane was purchased from Millipore company, (United States). Enhanced chemiluminescence kit (ECL) reagent was obtained from ThermoFisher Scientific, Ltd, (United States). Colchicine and LY294002 were obtained from MedChemExpress company (Shanghai, China) with the purity over 98%. Primary antibodies against Phospho-PI3K, Phospho-AKT, Phospho-eNOS, caspase3, GAPDH and antibodies against PI3K, AKT, eNOS, c-Caspase3 were purchased from ZEN-BIOSCIENCE company (Chendu, China).

### H9C2 Myocardial Cell Culture and Treatments

H9C2 cells were purchased from the National Collection of Authenticated Cell Cultures (Shanghai, China) and cultured in DMEM containing 10% FBS and 1% penicillin/streptomycin in CO_2_ incubator at 37°C and 95% relative humidity.

The H9C2 cells were pretreated with colchicine (0–5 μM) for 24 h, then the drug-containing medium was removed from the colchicine-treated cells and replaced by Krebs–Ringer Bicarbonate buffer, and cells were put into a modular incubator chamber saturated with 99.99% N_2_ and cultured in a normal incubator for the indicated time (0–16 h). Then the cells were subjected to reoxygenation by changing the DMEM back, and cultured under normoxia (21% O_2_) for 2 h. The H9C2 cells Hypoxia/Reoxygenation (H/R) model were established.

### Detection of Cell Viability

CCK-8 assays were carried out to detect the effect of colchicine on H9C2 cell activity. Briefly, the H9C2 cells were seeded into 96-well plates at the density of 3 × 10^3^ cells in culture medium. After processing the cells as previously described, 10 μL of CCK-8 was then added to each well and placed the 96-well plate in the incubator for 1 h. The absorbance values of each well were determined spectrophotometrically at 450 nm using a microplate reader (Bio-Tek, United States). Each experiment was repeated 5 times.

### Detection of Apoptosis

H9C2 cell apoptosis was detected by flow cytometry (Beckman Coulter Inc, Atlanta, Georgia, United States). The cells were trypsinized using 0.25% trypsin. The H9C2 cells in suspension (1 × 10^6^ cells) were extracted and washed using phosphate-buffered saline. The cells were resuscitated with 100 μL of binding buffer, mixed with 5 μL of Annexin V-FITC and 10 μL of PI, and incubated at room temperature in dark for 15 min. The samples were assessed using flow cytometer analysis. Each experiment was repeated 3 times.

### Western Blot Analysis

To determine anti-MIRI effects of colchicine through caspase3 and PI3K/AKT/eNOS signaling, protein phosphorylation was measured by western blotting assay. Briefly, H9C2-cell lysates were prepared in RIPA Lysis buffer after drug administration. The protein concentrations were analyzed by the BCA protein assay kit. Protein extracts were separated by 8–15% SDS-PAGE according to different molecular weights, and then transferred onto PVDF membrane. The membranes were blocked with 5% nonfat dry milk in TBST for 1 h at room temperature, and were then incubated overnight at 4°C with the following primary antibodies: p-PI3K (1:1,000), *p*-Akt (1:1,000), *p*-eNOS (1:1,000), Caspase-3 (1:1,000) and GAPDH (1:1,000). The membranes were then incubated for 1 h with goat anti-rabbit IgG-HRP (1:5,000) as the secondary antibody. Antibody-bound protein was detected by ECL reagent. GAPDH served as the internal control. ImageJ Java 1.8.0 software (update time: 2021-09-28) (NIH, United States) was used to analyze the imaging map and calculate the ratios of PI3K, AKT, eNOS and Cascapse3 proteins in each group.

### Statistical Analysis

All the data are expressed as the mean ± SD. The statistical analysis was performed with SPSS version 21.0 (update time: 2020) (IBM Corporation, Armonk, NY). For multiple comparisons, one-way analysis of variance (ANOVA) was performed, and comparisons between two groups were analyzed using an LSD test. A value of *p* < 0.05 was regarded as statistically significant.

### Ethics Statements

This study is not animal study, human study and have no identifiable human images/data.

## Results

### Active Components and Potential Targets of Colchicine on MIRI

**FIGURE 2:**
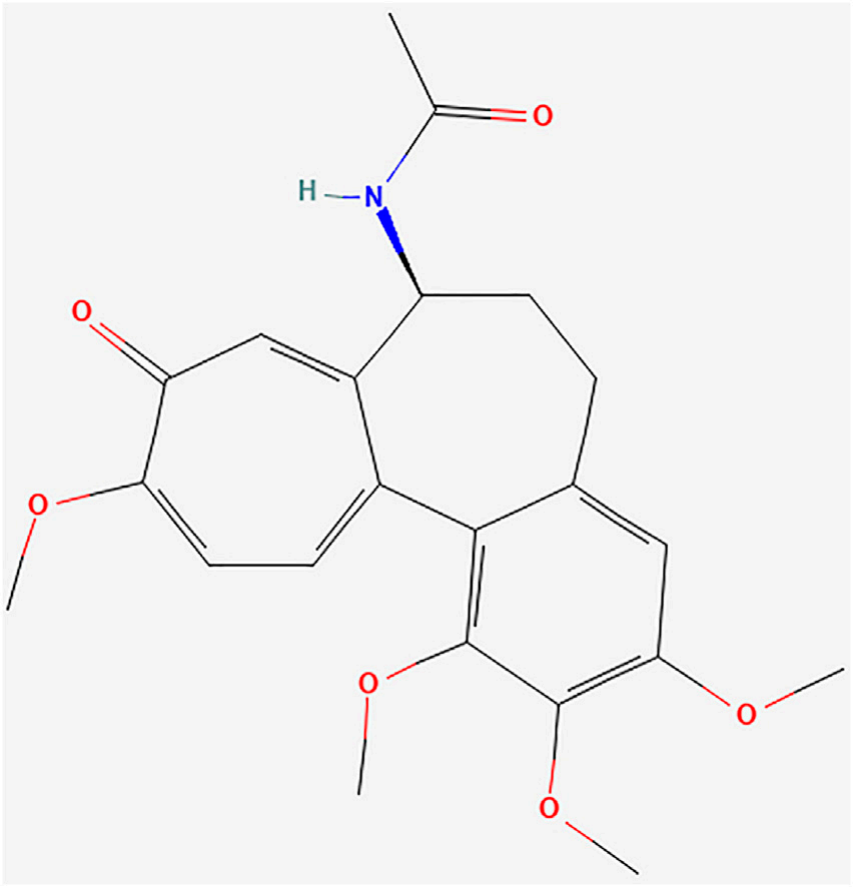
Chemical structure of Colchicine (PubChem CID: 6167).

**FIGURE 3:**
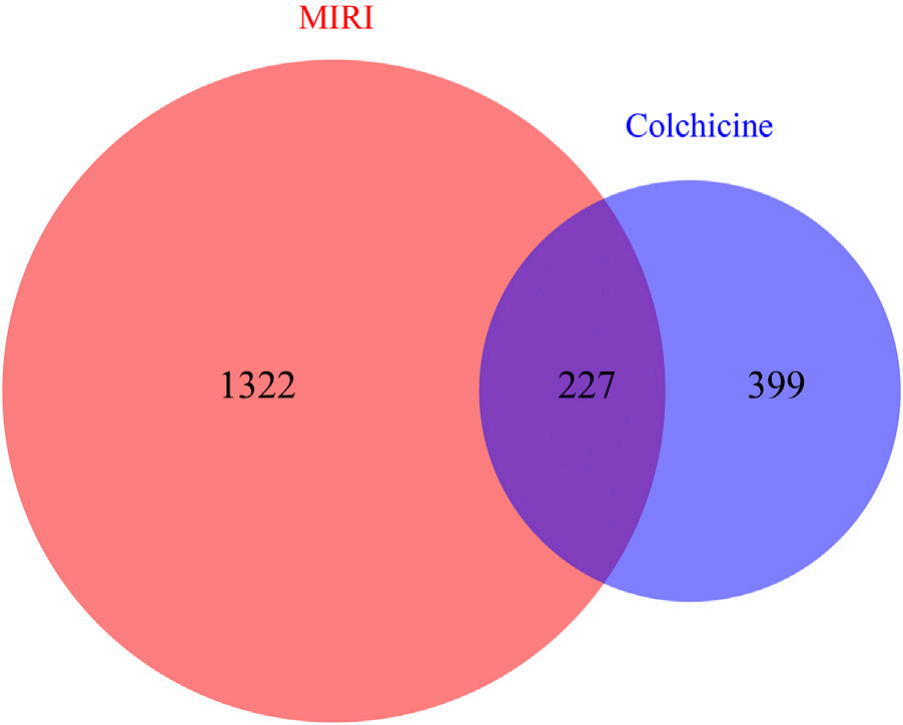
Venn diagrams showing the number of shared and unique targets by Colchicine and MIRI were represented. A total of 227 targets of colchicine with therapeutic potential for MIRI were identified.

The molecular formula of colchicine is C22H25NO6, and its molecular structure is shown in Figure 2. We searched the PharmMapper, Swiss Target Prediction, CTD, and BATMAN-TCM database, and a total of 626 target genes for colchicine were obtained by UniProt gene annotation simultaneously (Supplementary Table S1 **)**. For MIRI, we collected 1,549 disease targets through OMIM, GeneCards, and TTD (Supplementary Table S2). By matching the targets of colchicine with MIRI related targets, 227 cross-genes were derived, which were potential targets of colchicine in treating MIRI **(** Figure 3 **)**.

### Conversion of Target Proteins Into Network and Analysis

**FIGURE 4:**
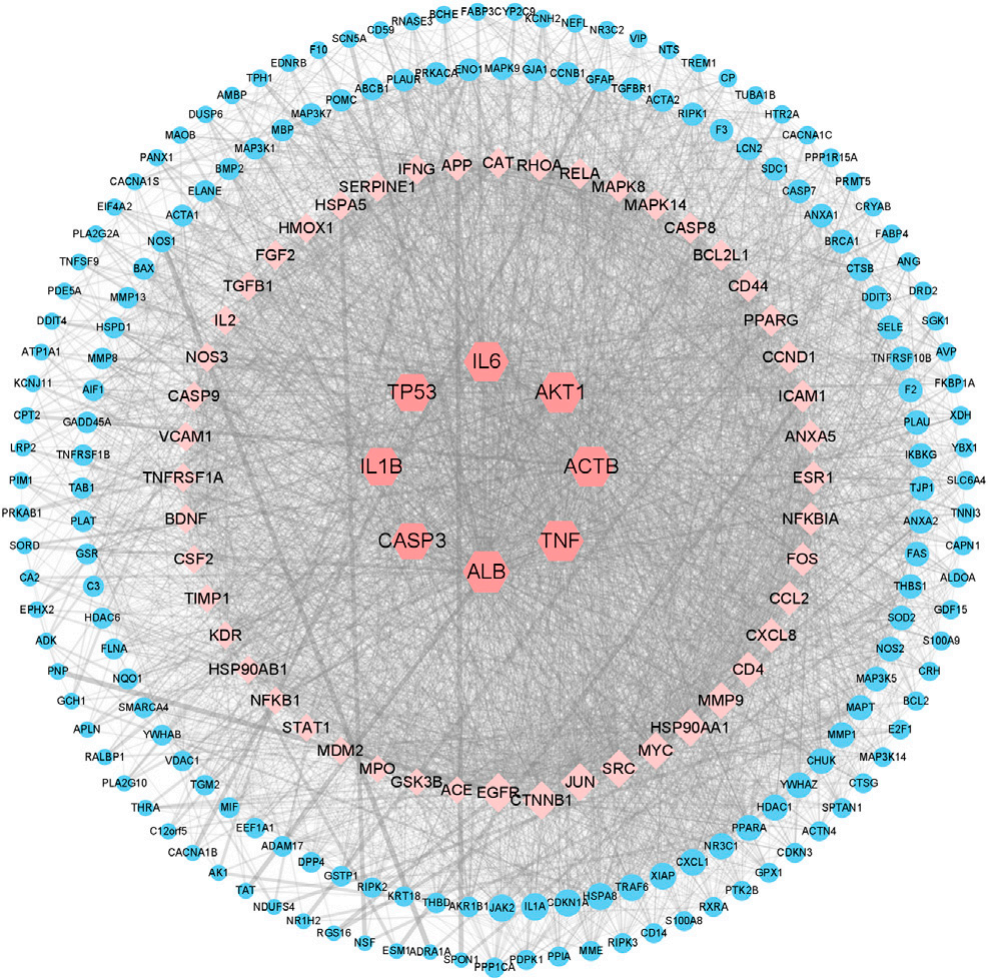
Network pharmacology revealed the targets characteristics of colchicine with therapeutic potential for MIRI. A protein network of known targets of colchicine with therapeutic potential for MIRI was generated by Cytoscape.

We uploaded a total of 227 cross-genes into the STRING database for PPI analysis. Then we imported the results from STRING database into Cytoscape to estimate the topological properties of PPI network. From Supplementary Figure S1, a total of 227 nodes and 4,709 edges were acquired. We selected 56 core target genes based on the Q3 of node degree (61), included ALB, TNF, ACTB, AKT1, IL6, TP53, IL1B, CASP3, et al. Figure 4 showed the topological properties results of these 56 core target genes with no isolated nodes. Nodes represented proteins, and each edge represented the interaction relationship between proteins. The more lines, the greater the correlation degree. The average clustering coefficient is 0.4931, average degree is 89.6250, average shortest path length is between 1.3097~1.7655, and betweenness centrality is between 0.0013~0.0747. According to our results, it is suggested that ALB, TNF, ACTB, AKT1, IL6, TP53, IL1B, CASP3, may be the key targets of colchicine in the treatment of MIRI.

### Gene Ontology Enrichment Analysis

**FIGURE 5:**
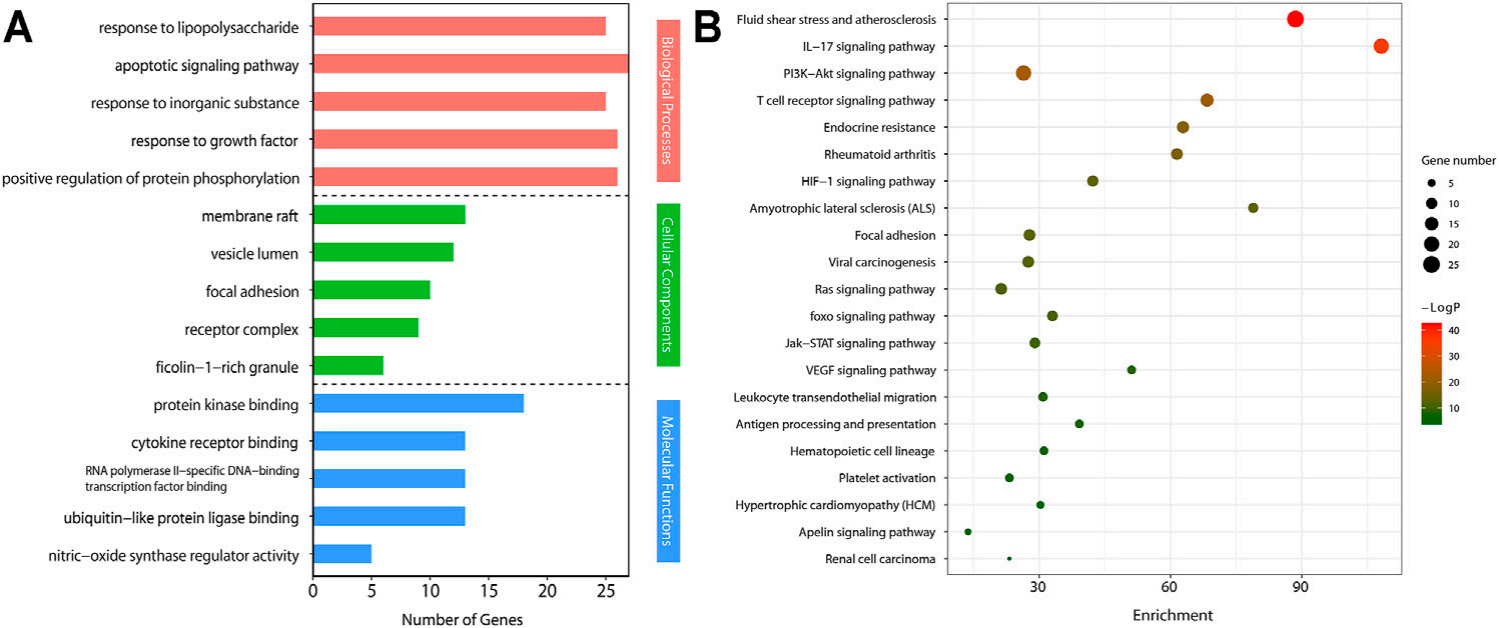
Gene Ontology (GO) analyses and Kyoto Encyclopedia of Genes and Genomes (KEGG) pathways by Metascape.**(A)** GO enrichment analysis of target genes. The number of GO entries in the functional categories of biological process (BP), cell composition (CC), and molecular function (MF) (*p* < 0.05).**(B)** KEGG pathways of target genes (*p* < 0.05).

We imported the selected potential 56 core target genes into the Metascape for GO analysis. A total of 98 biological processes were enrichment, such as response to lipopolysaccharide, apoptotic signaling pathway, response to inorganic substance, and et al. A total of 14 cellular component were enrichment, such as membrane raft, vesicle lumen, focal adhesion, and et al. A total of 23 molecular function were enrichment, such as protein kinase binding, cytokine receptor binding, RNA polymerase II-specific DNA-binding transcription factor binding, and et al. (Figure 5A).

### KEGG Pathway Enrichment Analysis

**FIGURE 6:**
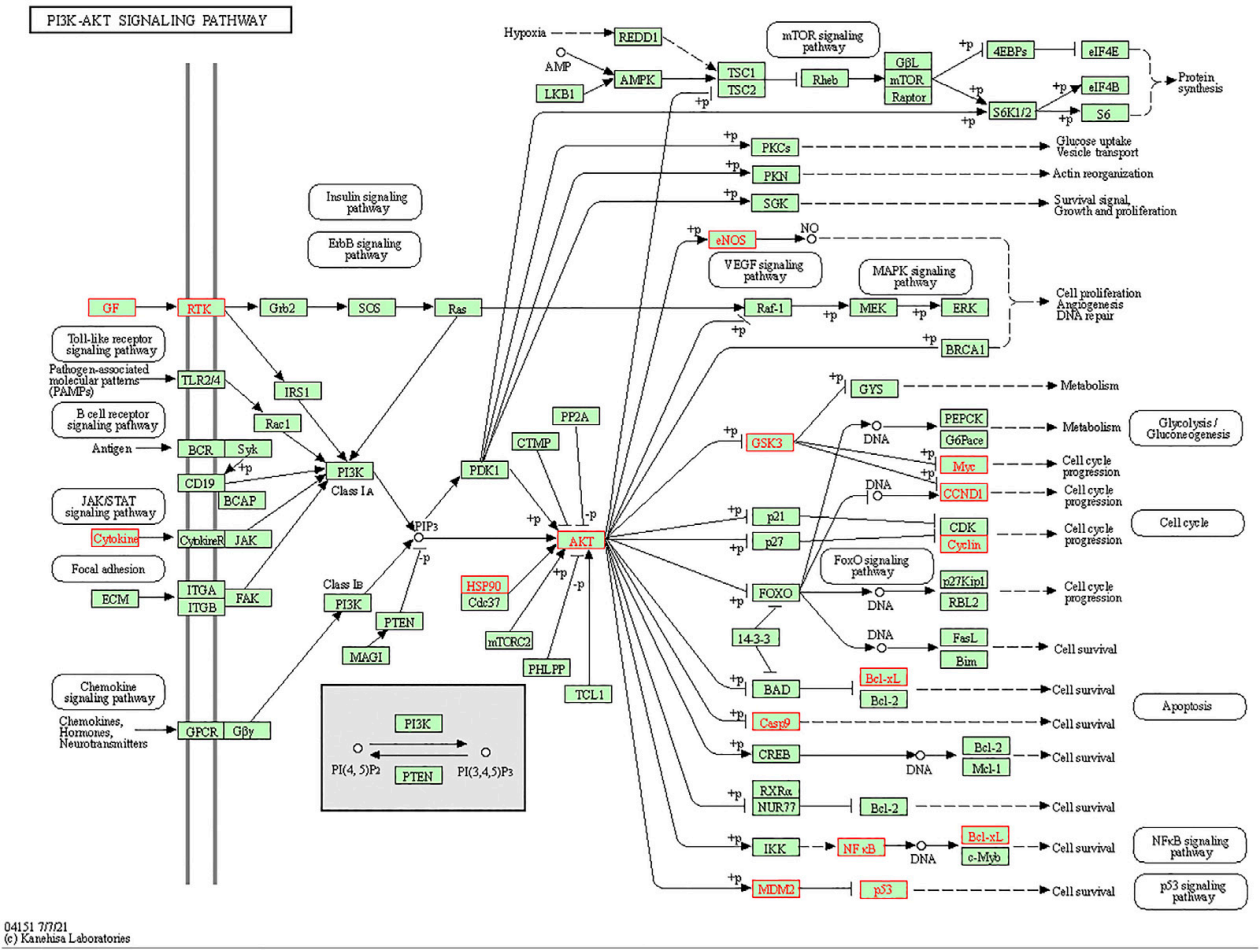
Screening of PI3K/AKT signaling pathway in apoptosis. The red marked nodes represent overlapping targets of colchicine and MIRI.

We conducted KEGG pathway enrichment analysis on potential 56 core target genes using Metascape and screened out 21 pathways. Figure 5B showed these 21 pathways, such as Fluid shear stress and atherosclerosis, IL-17 signaling pathway, PI3K-Akt signaling pathway, T cell receptor signaling pathway, and et al. As shown in Figure 5B, colchicine may play an anti-MIRI role by regulating cascade reactions of multiple signaling pathways. Interestingly, consistent with the results of the previous GO functional annotation, the KEGG pathway also enriched the apoptotic pathways. It is known that apoptosis plays an important role in MIRI injury of myocardial cells. Therefore, it is suggesting that colchicine may protect ischemic myocardium by inhibiting apoptosis in myocardial cells. Based on the enriched pathway of apoptosis, the PI3K/AKT signaling pathway (https://www.kegg.jp/kegg/) was obtained through further screening in the apoptotic mechanism (Figure 6).

### Molecular Docking Analysis

**FIGURE 7:**
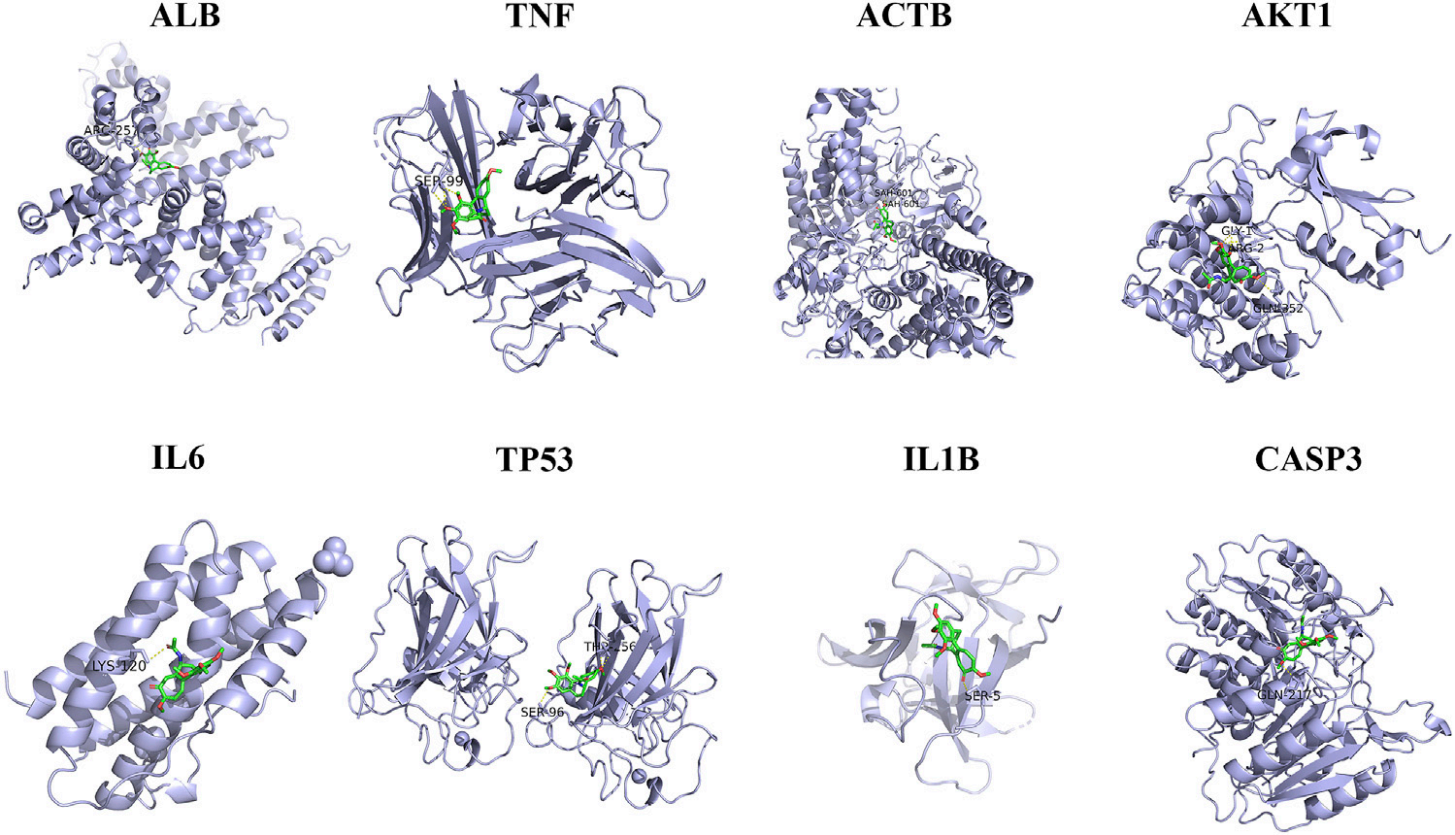
Structural interactions of colchicine and key target receptors.

Molecular docking analysis was conducted to evaluate the binding affinity of colchicine with key target proteins. In order to evaluate the biological activity of colchicine, the docking simulation of colchicine and the key target proteins were implemented by molecular docking analysis. As can be seen from Figure 7 and Table 1, the results showed that docking scores of colchicine with ALB, TNF, ACTB, AKT1, IL6, TP53, IL1B and CASP3 ranged from -6.1 to -7.6, and all the hub proteins showed better binding affinity with colchicine. Most of these hub proteins are involved in cell apoptosis.

**TABLE 1:** The docking scores of colchicine with key proteins.

Tagret	Drug	PDB ID	Docking score
ALB	Colchicine	1E7E	−7.6
TNF	—	6OOY	−6.6
ACTB	—	6ICV	−7.0
AKT1	—	3CQU	−6.5
IL6	—	1ALU	−6.1
TP53	—	6SHZ	−6.5
IL1B	—	1HIB	−6.2
CASP3	—	1CP3	−6.6

### Result of CCK-8 Cell Viability Assay

**FIGURE 8:**
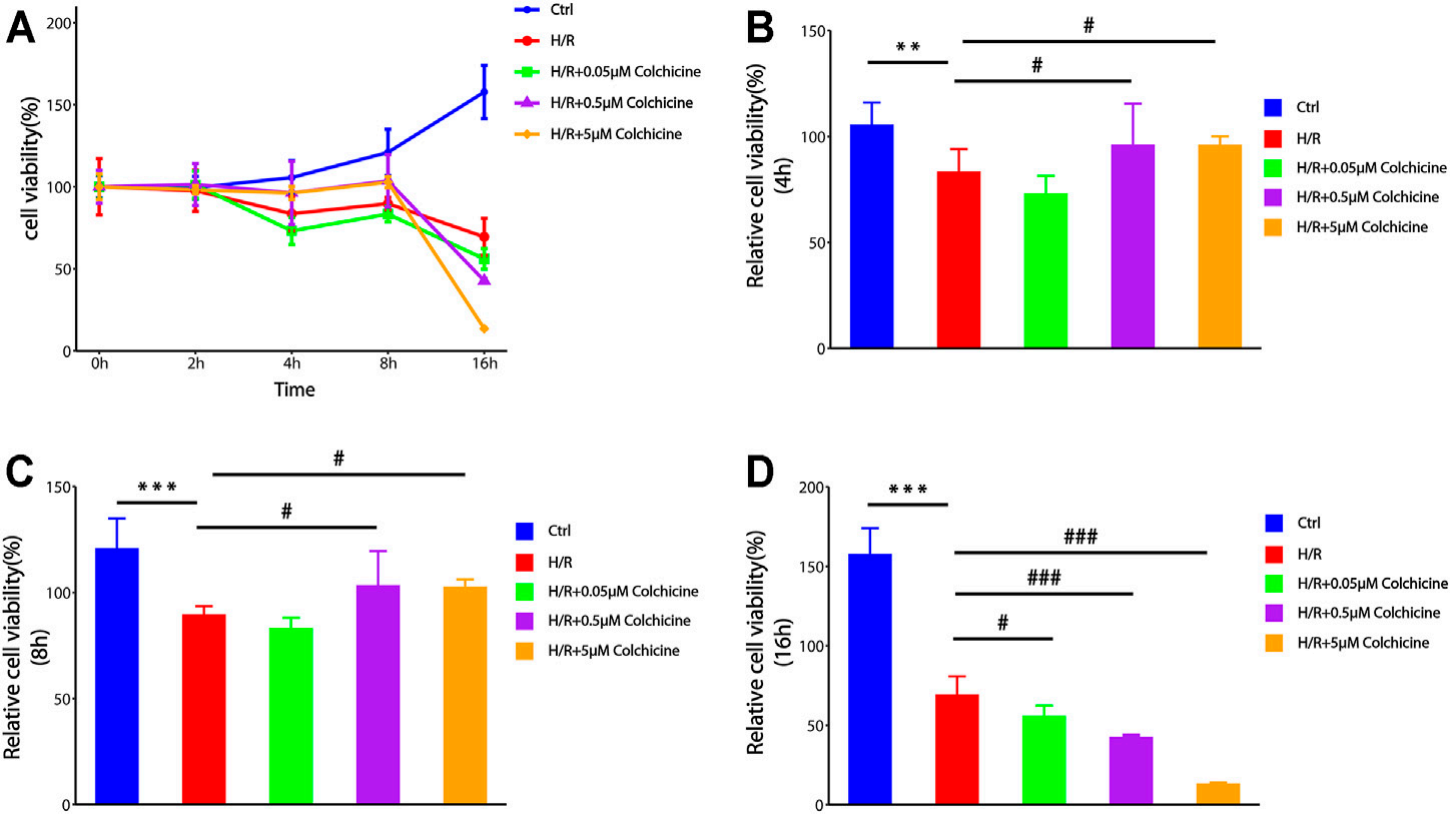
The effect of different concentrations of colchicine and different time of H/R-damaged on the viability of H9C2 cell by CCK-8 assays.**(A)** Optimum concentration of colchicine in appropriate time H/R-damaged was determined by CCK8 assay.**(B–D)** The effect of different colchicine concentrations on H9C2 cell viability after 4 h/8 h/16 h H/R injury (***p < 0.001, **p < 0.01 compared with control; #*p* < 0.05, ###*p* < 0.001 compared with H/R group).

To determine the effect of colchicine on H9C2 cells, we used different concentrations of colchicine (0.05, 0.5, 5 μM) to stimulate H9C2 cells and detected the cell viability by CCK8 assay. The cell viability was also measured after incubation with various time of H/R (0, 2, 4, 8, 16 h). From Figure 8, we found that groups with 0.5 μM or 5 μM colchicine have significantly higher cell viability than others hypoxia groups before 8 h, and the results of 5 μM colchicine group were more stable (the SD was smaller). We also observed that there was a significant decrease in cell viability of 0.5 and 5 μM colchicine groups at 16 h. Maybe it’s the toxic effects of colchicine. Therefore, we choose 5 μM as the optimum concentration of colchicine to anti-MIRI at 8 h.

### Result of Apoptosis Detection

**FIGURE 9:**
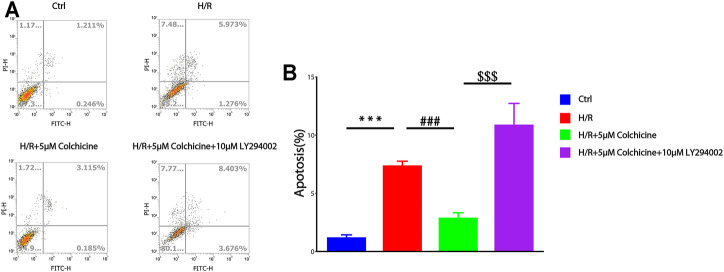
Flow cytometry was used to detect the apoptosis-inducing effect of colchicine on H9C2 cells. **(A)** the apoptotic effects of colchicine was measured through flow cytometry.**(B)** The quantitative results were analyzed. The data were expressed as mean ± SD (***p < 0.001 compared with control; ###*p* < 0.001 compared with H/R group; $$$*p* < 0.001 compared with colchicine treated alone).

CCK-8 assay results suggested that colchicine can protect H9C2 cell form H/R injury. To determine whether this anti-MIRI effect was related to apoptosis, flow cytometry was used to detect the apoptosis-inducing effect of colchicine on H9C2 cells and the percentage of apoptotic cells was also detected by Annexin V-FITC and PI double staining. As shown in Figure 9, the apoptosis rate was increased in 6.182%, which is significantly when cells were treated with H/R compared with the controls (1.214%). However, colchicine with 5 μM caused a significant reduction (4.490%) of early apoptotic cells as compared with the H/R only groups. Additionally, LY294002 (PI3K inhibitor) significantly attenuated the effects of colchicine, which can be observed from the apoptotic rate increased to 7.993%. These results suggested that colchicine plays an antiapoptotic role partly on H/R injury of H9C2 cell.

### Result of Western Blot Analysis

**FIGURE 10:**
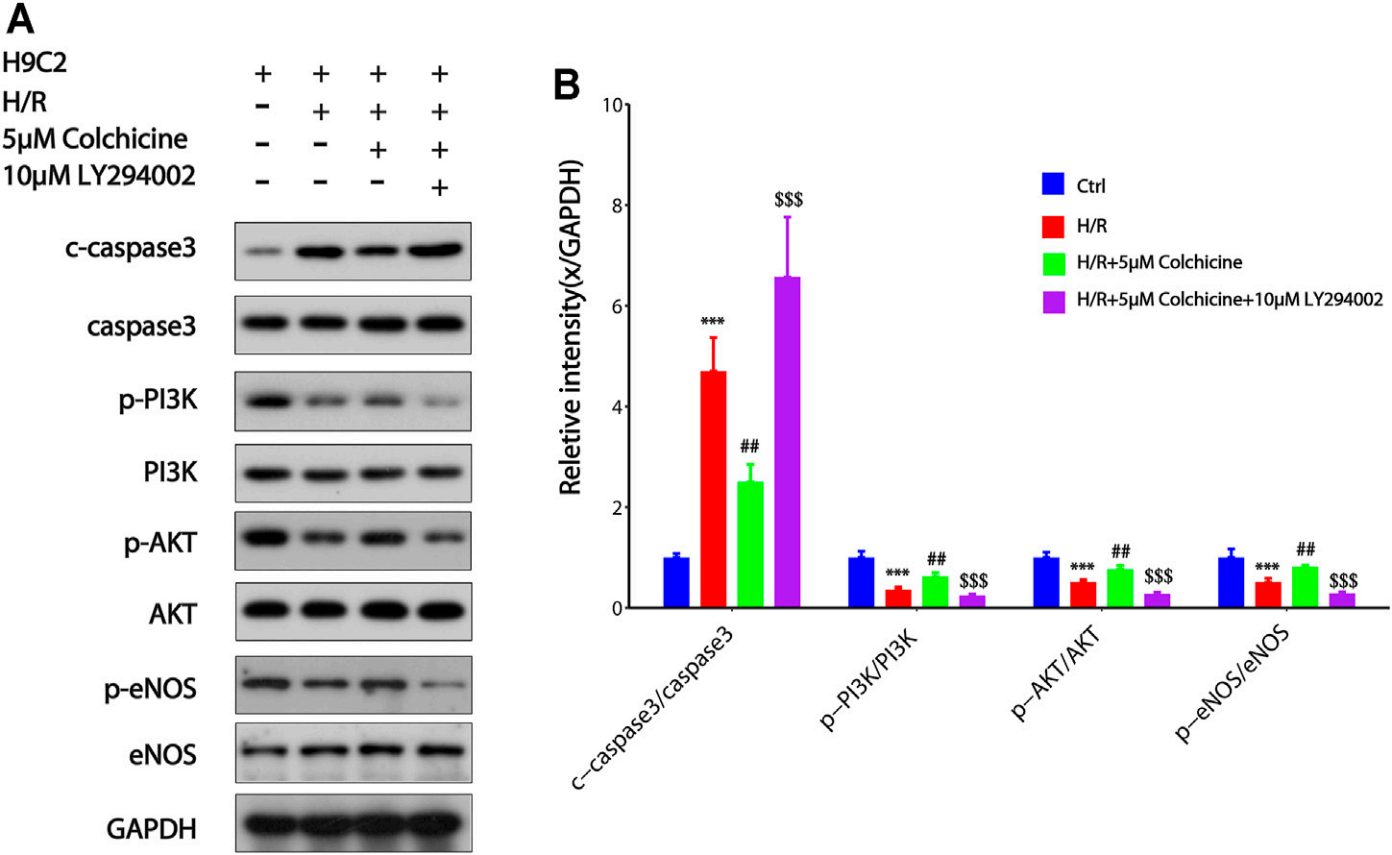
Colchicine inhibited MIRI-induced apoptosis of H9C2 by regulating the PI3K/AKT/eNOS pathway.**(A)** Protein expression of p-PI3K, *p*-AKT, *p*-eNOS, and Caspase-3 were detected by western blot analysis;**(B)** Protein content was quantified using densitometric analysis with GAPDH as internal control. The values of control group were set as one. Data were expressed as mean ± SD (***p < 0.001 compared with control; ##*p* < 0.01 compared with H/R group; $$$*p* < 0.001 compared with colchicine treated alone).

PI3K/AKT is one of the main signal transduction pathways involved in cell proliferation and apoptosis, and eNOS is a downstream of PI3K/AKT which related to the anti-MIRI effect of colchicine base on the result of GO enrichment and KEGG enrichment (Zhang et al., 2019). To further study the mechanisms underlying the antiapoptotic effects of colchicine and the effects of colchicine on PI3K/AKT/eNOS signaling pathway, we detected the apoptosis-related protein caspase3 and the key proteins of PI3K, AKT, eNOS in the signaling pathway. From Figure 10, it could be seen that after H/R injury in H9C2 cell, phosphorylation levels of PI3K, AKT, and eNOS proteins were significantly decreased compared with those in the control group (*p* < 0.001). Compared with the H/R group, 5 μM colchicine observably increased the phosphorylation levels of PI3K, AKT, and eNOS (*p* < 0.01). Simultaneously, c-Caspase3 was significantly increased expression in H/R treated H9C2 cell (*p* < 0.001), however, markedly reduced by 5 μM colchicine treatment (*p* < 0.01). Moreover, the use of LY294002 (PI3K inhibitor) significantly prevented the effects of colchicine (*p* < 0.001), which can be shown from the protein expression of p-PI3K, *p*-AKT, *p*-eNOS and c-Caspase-3. Our results demonstrated that colchicine can inhibited MIRI induced apoptosis of H9C2 cell by activating the PI3K/AKT/eNOS pathway.

## Discussion

MIRI is a common pathophysiological injury in ischemic heart disease, shock, extracorporeal circulation, cardiovascular surgery and other diseases or treatments. It causes damage to tissues, organs, etc. through an excessive cascade reaction (Gao et al., 2012). Moreover, together with the injury caused by ischemia, it determines the final region of myocardial infarction and the process of ventricular remodeling, and eventually develops into heart failure (Herr et al., 2020). At present, endogenous protective methods are widely used to reduce reperfusion injury and improve the prognosis of patients with acute myocardial infarction, for example, ischemic preconditioning, ischemic post-conditioning and pharmacological post-conditioning (Wu et al., 2018). Nevertheless, there are few effective drugs for the treatment of MIRI clinically. Therefore, finding a resultful drug to treat MIRI makes sense (Bellis et al., 2020). Network pharmacology is an emerging discipline based on systems biology, which is used to analyze biological system networks to select specific signal nodes, so that the potential interaction between drugs and targets can be accurately determined (Barabasi et al., 2011; Zhao et al., 2016). For these reasons, there is no doubt that network pharmacology is one of the efficacious strategies for discovering new drugs (Hopkins, 2008). This study discovered the potential therapeutic targets and pathways of colchicine through network pharmacology, and explored the mechanism of colchicine on MIRI. Our works provide new ideas for understanding the pharmacological mechanisms of drugs from the perspective of the network.

Colchicine has various biological activities such as anti-inflammatory, anti-hepatic fibrosis, and enhance the effects of sympathomimetic drugs and so on (Leung et al., 2015). The beneficial effects of colchicine on cardiovascular disease have been widely reported in recent studies (Roubille et al., 2014; Martinez et al., 2018; Akodad et al., 2020). Previous reports had shown that colchicine has a certain protective effect on ischemic myocardium, which can reduce the coronary plaque and the area of myocardial infarction in patients with myocardial infarction by inhibiting inflammation response (Nidorf et al., 2019; Nidorf et al., 2020). However, the potential regulatory targets and pharmacological mechanisms of colchicine in MIRI injury remain unclear. In this study, 227 overlapping genes related to colchicine were identified by connecting targets of colchicine with targets of disease. the PPI network analysis demonstrated that the targets linked to MIRI were ALB, TNF, ACTB, AKT1, IL6, TP53, IL1B, CASP3 and these targets showed nice affinity with colchicine in molecular docking experiments, we found that most of the hub targets are involved in external or internal apoptotic pathways. The results of GO analysis and KEGG pathway enrichment demonstrated that the anti-MIRI effect of colchicine involves in apoptotic signaling pathway and the PI3K/AKT signaling pathway was obtained through further screening in the apoptotic mechanism. Previous studies have extensively reported the beneficial effects of inhibition of cardiomyocyte apoptosis on MIRI (Tao et al., 2011; Konstantinidis et al., 2012). However, the effect of colchicine on cardiomyocyte apoptosis induced by MIRI has not been reported. Therefore, network pharmacology can help us understand the mechanism of colchicine in the treatment of MIRI from a general view and lays a good foundation for further discussion of the anti-MIRI pharmacological target and molecular mechanism of colchicine.

Based on KEGG pathway enrichment and the result of molecular docking in the core targets, we chose the key proteins of PI3K/AKT/eNOS signaling pathway and the apoptosis-related protein Caspase-3 for validation the mechanisms underlying the anti-MIRI effects of colchicine. The PI3K/Akt signal pathway can regulate the migration, proliferation, differentiation, apoptosis and metabolism of various types of cells, which is connected to cardiomyocyte apoptosis (Brand et al., 2019; Cui et al., 2020). PI3K is activated and generates phosphatidylinositol 3,4,5-trisphosphate, which induces the phosphorylation of AKT (*p*-AKT), *p*-AKT then activates endothelial nitric oxide synthase (eNOS) and produces NO, NO produced by eNOS activation has been shown to play an important role in the process of MIRI (Cohen et al., 2006; Ying et al., 2015). CASP3, as one of the most concerned proteins in caspase family, can catalyze the specific cleavage of a variety of key cell proteins, which is one of the most critical apoptotic protease in the process of apoptosis (Miura, 2011). Activation of caspase-3 is a common ultimate signal in response to factors that induce apoptosis, such as plays an important role in the apoptosis of cardiomyocytes (Gundapaneni et al., 2017).

Our present data provide ample evidence to support the protective role of colchicine in MIRI injury through reducing apoptosis. In this study, we found that colchicine could systematically and significantly inhibit the apoptosis cells from H/R injure. Additionally, LY294002 (PI3K inhibitor) significantly attenuated the effects of colchicine. Our results demonstrated that colchicine markedly enhanced the decreased levels of Caspase-3 in myocardial injure model induced by H/R. Then, Western blot results further confirmed that colchicine inhibited H/R induced cardiomyocyte apoptosis by regulating the PI3K/AKT/eNOS pathway. After PI3K/AKT/eNOS signal pathway was specifically blocked (LY294002 group), cardiomyocyte apoptosis induced by ischemia-reperfusion was aggravated. This further suggested that colchicine plays a protective role against MIRI through the apoptosis pathway. R. Singhal et al. found that the activation of PI3K/AKT/eNOS pathway induced by colchicine could reduce apoptosis, inflammation and fibrosis in heart failure cardiomyocytes, which may in part contribute to the improvement of cardiac function (Singhal et al., 2014). This result is consistent with the PI3K/AKT/eNOS signaling pathway and key targets related to apoptosis obtained in our study.

## Conclusion

In summary, we performed network pharmacology and experimental evaluation to reveal the pharmacological mechanism of colchicine against MIRI. Moreover, a further *in-vitro* validation experiment illustrated that colchicine substantially inhibited apoptosis of myocardial cell via regulating PI3K/Akt/eNOS signaling pathway and inhibiting the expression of pro-apoptotic protein Caspase-3. Our findings may guide further pharmacological investigations on the therapeutic effects of colchicine on MIRI and the discovery of new drugs for MIRI treatment. However, this study still has some limitations. Network pharmacology approaches await further improvements, and it is necessary to conduct *in vivo* experimental study to verify the efficacy and mechanism of colchicine in the treatment of MIRI in our future research.

## Data Availability

The original contributions presented in the study are included in the article/Supplementary Material, further inquiries can be directed to the corresponding authors.
