# Supplementary figures and images for: Network Pharmacology-Based Investigation and Experimental Exploration of the Antiapoptotic Mechanism of Colchicine on Myocardial Ischemia Reperfusion Injury
Source: Front Pharmacol. 2021 Dec 16;12:804030. doi: 10.3389/fphar.2021.804030 (PMC8716846; doi:10.3389/fphar.2021.804030)

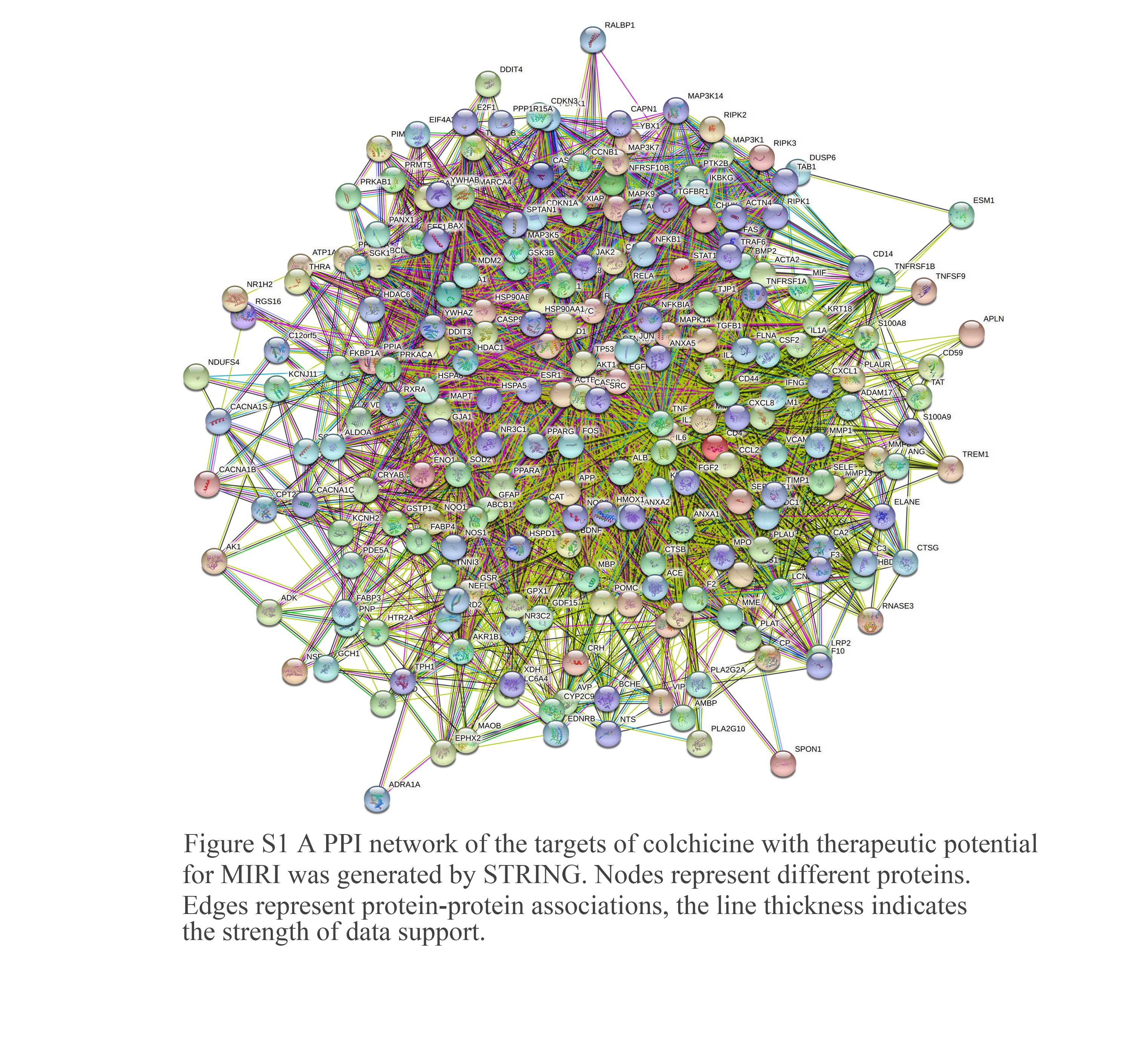

Supplement: Supplementary file 3 [file Image1.jpg]
